# Gene divergence of homeologous regions associated with a major seed protein content QTL in soybean

**DOI:** 10.3389/fpls.2013.00176

**Published:** 2013-06-05

**Authors:** Puji Lestari, Kyujung Van, Jayern Lee, Yang Jae Kang, Suk-Ha Lee

**Affiliations:** ^1^Department of Plant Science, Research Institute for Agriculture and Life Sciences, Seoul National UniversitySeoul, Korea; ^2^Indonesian Center for Agricultural Biotechnology and Genetic Resources Research and DevelopmentBogor, Indonesia; ^3^Plant Genomics and Breeding Institute, Seoul National UniversitySeoul, Korea

**Keywords:** genome duplication, QTL, seed protein content, soybean, sequence divergence

## Abstract

Understanding several modes of duplication contributing on the present genome structure is getting an attention because it could be related to numerous agronomically important traits. Since soybean serves as a rich protein source for animal feeds and human consumption, breeding efforts in soybean have been directed toward enhancing seed protein content. The publicly available soybean sequences and its genomically featured elements facilitate comprehending of quantitative trait loci (QTL) for seed protein content in concordance with homeologous regions in soybean genome. Although parts of chromosome (Chr) 20 and Chr 10 showed synteny, QTLs for seed protein content present only on Chr 20. Using comparative analysis of gene contents in recently duplicated genomic regions harboring QTL for protein/oil content on Chrs 20 and 10, a total of 27 genes are present in duplicated regions of both Chrs. Notably, 4 tandem duplicates of the putative homeobox protein 22 (HB22) are present only on Chr 20 and this *Medicago truncatula* homolog expressed in endosperm at seed filling stage. These tandem duplicates could contribute on the protein/oil QTL of Chr 20. Our study suggests that non-shared gene contents within the duplicated genomic regions might lead to absence/presence of QTL related to protein/oil content.

## Introduction

Since soybean [*Glycine max* (L.) Merrill] seed is a good source of protein and oil, it is grown widely throughout the world for its numerous uses, such as various edible products, animal feed and potential industrial applications (Vuong et al., 2007; Van et al., 2008; Kim et al., 2012). Although the wild soybean (*G. soja* Sieb. and Zucc.), an undomesticated form of the current soybean, is distributed in East Asia, including China, Taiwan, Russian Far East, the Korean Peninsula, and Japan, the origin or domestication site of soybean is still in controversy (Boerma and Specht, 2004; Van et al., in press). After soybean is introduced into Central and South America in the mid-1900's via North America in 1765, soybean becomes one of the major economically valuable crops in terms of the world's total production (Vuong et al., 2007; Stupar and Specht, 2013).

Although high seed protein content directs soybean products having greater nutritional value, the complexity of soybean genome made difficulty for rapid development of strategies in soybean breeding programs. Before the genomic era, SoyBase (http://soybase.org) is the main resource for quantitative trait loci (QTLs) for various traits and linkage map with 20 soybean chromosomes (Chrs). Also, classical, allozyme and other genetic markers such as restriction fragment length polymorphism (RFLP), amplified fragment length polymorphism (AFLP), simple sequence repeats (SSRs) and single nucleotide polymorphism (SNP), are publically available. Starting with the genome sequences of the *G. max* cultivar (Williams 82, Schmutz et al., 2010), the tremendous amount of sequence information generated by resequencing of *G. max* accessions and *G. soja* against the reference genome (Kim et al., 2010; Lam et al., 2010) would be more feasible for soybean improvement.

This review aims to introduce soybean genome complexity in terms of genome duplication and the recent researches of the major QTLs for seed protein content and to suggest gene divergence in homeologous regions related to this QTL with respect to genome duplication between two soybean Chrs 20 and 10.

## Soybean genome structure

Genome duplication is a key process in the evolution of many lineages in flowering plants (Zhu et al., 2005; Flagel and Wendel, 2009). Following whole genome duplication, small-scale duplications are arisen from unequal crossing over and chromosomal anomalies (Freeling, 2009). After crossing over, several kinds of mechanisms including translocation, inversion, deletion and duplication play a considerable role during small duplications (Pagel et al., 2004). If whole genome duplications tend to increase the dosage gene simultaneously, small-scale duplications (tandem and segmental duplications) result in genes out of balance to maintain proper balance (Edger and Pires, 2009).

**Figure 1:**
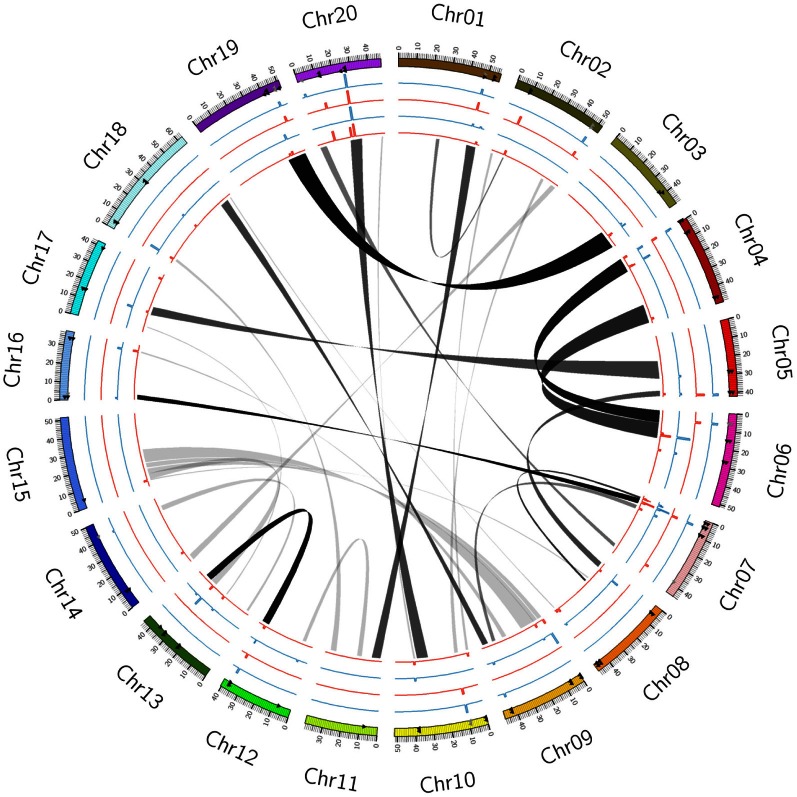
**Circular map showing homeologous relationships among 20 soybean chromosomes based on QTLs associated with protein and oil content in recently duplicated segments**. Chr01–Chr20 denote chromosome number of the soybean genome. The scale of 0–50 kb represents physical locations. Gray ribbons represent as duplicated positions of the *G. max* genome after similar duplicated regions are grouped as bundles. Circular lines from the inner to the outer: 1, LOD of protein QTL (red); 2, *R*^2^ of protein QTL (blue); 3, LOD of oil QTL (red); 4, *R*^2^ of oil QTL (blue).

The moderately large soybean genome (1.1 Gb) with ancient and recent duplications demonstrates that soybean genome is complex (*Glycine max* v1.0 at http://www.phytozome.net/soybean.php). The second round of soybean whole genome duplication occurred approximately 13 million years ago and this polyploidy event contributes to the soybean genome structure ranging from near-identical, rather divergent to latter more divergent, leading to dynamic and massive genome rearrangement (Wendel and Doyle, 2005; Schmutz et al., 2010). The predicted number of coding genes in soybean is higher than that of Arabidopsis and grape, possibly due to the genome duplication events in soybean's history (Sterck et al., 2007; Cannon and Shoemaker, 2012). Based on the homeologous relationships determined by genome assembly of integrated data from recently duplicated genomic segments (http://www.phytozome.net; http://www.soybase.org), homeologous blocks of duplicated segments were found in all 20 Chrs (Figure 1, gray ribbons). Multiple blocks on more than two Chrs indicate homoeologous retention and chromosomal rearrangements (Schmutz et al., 2010).

Various gene duplications should be useful as subject to evolutionary divergence because the mode of duplication can influence evolutionary outcomes and plant specific traits are affected by functional gene duplication (Kaessmann, 2010; Cannon and Shoemaker, 2012; Yang and Bharti, 2012). A large impact of segmental duplications has been reported on the evolution of genes involved in phenotypic traits such as disease resistance and developmental process. QTLs associated with corn earworm resistance, Sclerotinia stem rot, soybean cyst nematode, seed-related traits (size, weight, and yield) and contents of protein, oil and sucrose were conserved across homeologous genomic regions after genome duplication (Shin et al., 2008; Kim et al., 2009). Therefore, the integration of soybean genomics with relative phenotypic trait resources should facilitate the identification of homeologous chromosomal rearrangements and new duplicate gene copies and help to identify informative QTLs related to desirable traits in soybean.

## QTLs for seed protein/oil content

Seed protein content has been investigated extensively in many soybean breeding programs (Helms and Orf, 1998; Cober and Voldeng, 2000; Panthee et al., 2005). Since seed protein content is determined by the interaction of various genetic loci with environmental factors, traditional soybean breeding has been assisted by extensive linkage map analyses, which have been conducted to identify QTLs for protein and oil contents with a range of genetic backgrounds and in different environments (Diers et al., 1992; Csanadi et al., 2001; Jun et al., 2008). Various soybean lines such as wild and cultivated soybeans and genotypes from different countries have also been used to explore seed protein QTLs (Sebolt et al., 2000; Csanadi et al., 2001; Jun et al., 2008).

From a large number of studies performed to identify QTLs for seed protein content in soybean, approximately 108 and 124 QTLs with various phenotypic variations have been correlated with the seed protein and oil content, respectively, and these were located on all of the soybean Chrs (http://soybase.org). Over 61 QTLs are associated with the protein content in 17 different soybean populations (Vuong et al., 2007). The seed composition traits may be associated with seed sucrose content in soybean and a QTL for seed sucrose content on Chr 20 made a phenotypic contribution of greater than 10%, which may be a major QTL with a pleiotropic effect (Maughan et al., 2000). Combined with soybean genomic analysis, the QTLs for protein and their related traits could facilitate the rapid selection of significant protein QTLs and the identification of candidate genes regulating seed protein content.

## A major QTL for seed protein content in respect to soybean genome duplication

Remarkable attention has been given to the major seed protein QTL mapped on Chr 20 [previously known as a linkage group (LG) I] because of the highest additive effect across many mapping populations and multiple environments (Brummer et al., 1997; Sebolt et al., 2000; Csanadi et al., 2001; Chung et al., 2003; Nichols et al., 2006). Accompanying with a reduced oil level, the application of marker-assisted selection to protein QTL on Chr 20 confirmed an increased production of protein in homozygous lines carrying alleles from a high protein parent (Diers et al., 1992; Yates et al., 2004) and the same correlation was also observed in different mapping populations using wild soybean as one of the parent (Brummer et al., 1997; Sebolt et al., 2000). The mapped QTL for protein and oil between Satt496 and Satt239 on Chr 20 had an additive effect of the PI 437088 alleles with increased protein level but reduced oil content (Chung et al., 2003). The near-isogenic line P-C609-45-2 was segregated at the smallest QTL interval on Chr 20, which corresponded to seed protein level (Nichols et al., 2006). Candidate genes identified by QTL analysis on Chr 20 have been associated with seed protein regulation and next-generation sequencing technology was also applied to an extensive investigation of the seed protein QTL on Chr 20 (Bolon et al., 2010; Severin et al., 2010). Although analyses of the linkage map and the major protein QTL on Chr 20 have been addressed using several approaches (Wang et al., 2006; Joseph, 2009; Qi et al., 2011), the regulation of seed protein content is not clear yet (Bolon et al., 2010). Furthermore, seed protein regulation may be related to soybean genome structure, such as gene duplication representing a primary source for gain of new gene function. It can be understood by whole genome and small-scale duplications facilitating an increase in biological complexity and evolutionary novelties (Van de Peer et al., 2009).

The recent genome duplication occurred frequently on many soybean Chrs, which is supported by the coincidence of several duplicate loci in the Chrs (Cannon and Shoemaker, 2012). Rearrangements of homeologous chromosomal regions are also observed in corresponding QTL regions related to both protein and oil traits. Based on Circos map, QTLs across duplicated regions were conserved, for example, Chr 4 vs. Chr 6 and Chr 3 vs. Chr 19 (Figure 1). Although Chr 20 shares high homology with the long arm of Chr 10, the major QTLs for seed protein content are only observed on Chr 20, not on its duplicated region of Chr 10 (Figure 1). It was reported that there is a close association between a QTL for seed composition in one member of a homeologous pair and a similar QTL on another duplicated pair (Shoemaker et al., 1996; Shin et al., 2008; Kim et al., 2009). However, protein and oil QTLs duplicated within interrelated homeologous regions showed rearrangement of the QTLs in homeologous pairs that occurred due to the recent duplication event (Figure 1; Shoemaker et al., 1996). The recent soybean genome structure shows that the major QTLs for soybean seed protein and oil are located mainly within not only homeologous regions (Chr 20) but also other homeologous regions (Chr 10) (Figure 1). The analysis of duplicated regions may suggest the rapid divergence of both regions at the chromosomal level (Chr 20 vs. Chr 10) (Picket and Meeks-Wagner, 1995).

## Comparison of duplicated regions associated with seed protein content

Duplicated regions in plant genome that contain genes may cause gene retention/loss, where polyploidy commonly contributes an expansion of gene copy (Cannon et al., 2004). Since subsequent duplication leads mutated genes to alter their functions, soybean genome duplication may also act on gene regulation (Shoemaker et al., 1996; Schmutz et al., 2010). The concordance of homeologous regions with QTLs for seed protein content support common roles, which homeologous loci and genetic redundancy inherited quantitatively (Shoemaker et al., 1996). However, it is assumed that the absence of the QTL in Chr 10 is derived from the absence of gene contents which could be decayed or from insertion of genes into Chr 20 after recent duplication event. The major QTL for seed protein contents, Prot 15-1, is associated with markers Satt239 and Satt496 on Chr20: 24,867,385..28,878,629 and its duplicated region is located on Chr10: 30,286,648..34,294,718. Among 81 genes in both duplicated regions, a total of 27 genes commonly identified in both regions and 19 and 35 genes were present only on Chr 20 and Chr 10, respectively (Table 1). Since genome duplication also gives a large impact on gene content and retention rate for balancing (Edger and Pires, 2009), the QTL for soybean seed protein could be a good clue to trace duplicated genes associated with seed protein content.

**Table 1:** **Gene divergence of duplicated regions between Chr 20 (24,867,385..28,878,629) and Chr 10 (30,286,648..34,294,718)**.

**Chromosome 20**	**Chromosome 10**	**Putative function**
−^*^	Glyma10g23750	Core-2/I-branching beta-1,6-N-acetylglucosaminyltransferase family protein
−	Glyma10g23790	Uricase/urate oxidase/nodulin 35, putative
−	Glyma10g23800	Concanavalin A-like lectin protein kinase family protein
−	Glyma10g23810	Exocyst subunit exo70 family protein A1
−	Glyma10g23840	Double Clp-N motif-containing P-loop nucleoside triphosphate hydrolases superfamily protein
−	Glyma10g23910	Polynucleotidyl transferase, ribonuclease H-like superfamily protein
−	Glyma10g24030	Glycosyl hydrolase superfamily protein
Glyma20g17960	Glyma10g24060	GTP-binding family protein
Glyma20g17990	−	Urease accessory protein D
Glyma20g18010	−	Pentatricopeptide (PPR) repeat-containing protein
Glyma20g18280	−	Terpene synthase 03
−	Glyma10g24080	Expansin B2
−	Glyma10g24100	Double Clp-N motif-containing P-loop nucleoside triphosphate hydrolases superfamily protein
−	Glyma10g24120	Expansin B2
−	Glyma10g24190	Leucine-rich repeat transmembrane protein kinase family protein
−	Glyma10g24200	AT1G21280.1
−	Glyma10g24270	Gibberellin 2-oxidase
Glyma20g18290	Glyma10g24340	AMMECR1 family
Glyma20g18420	−	F-box and associated interaction domains-containing protein
Glyma20g18440	Glyma10g24350	U2 small nuclear ribonucleoprotein A
Glyma20g18450	−	Homeobox protein 22
Glyma20g18460	−	Homeobox protein 22
Glyma20g18520	−	Homeobox protein 22
Glyma20g18540	−	Homeobox protein 22
Glyma20g18550	Glyma10g24360	P-loop containing nucleoside triphosphate hydrolases superfamily protein
−	Glyma10g24400	P-loop containing nucleoside triphosphate hydrolases superfamily protein
Glyma20g18620	Glyma10g24420	P-loop containing nucleoside triphosphate hydrolases superfamily protein
Glyma20g18860	Glyma10g24420	P-loop containing nucleoside triphosphate hydrolases superfamily protein
−	Glyma10g24430	Vesicle-associated membrane protein 726
Glyma20g18870	Glyma10g24540	Protein kinase superfamily protein
Glyma20g18890	Glyma10g24550	Ankyrin repeat family protein
−	Glyma10g24570	Ankyrin repeat family protein
Glyma20g18900	Glyma10g24580	RING/U-box superfamily protein
Glyma20g18970	Glyma10g24580	RING/U-box superfamily protein
Glyma20g18980	Glyma10g24590	Peroxisomal 3-ketoacyl-CoA thiolase 3
Glyma20g18990	−	hAT transposon superfamily
−	Glyma10g24600	DZC (Disease resistance/zinc finger/chromosome condensation-like region) domain containing protein
Glyma20g19000	Glyma10g24620	Potassium channel beta subunit 1
Glyma20g19200	Glyma10g24630	Pectin lyase-like superfamily protein
Glyma20g19210	Glyma10g24650	Inosine triphosphate pyrophosphatase family protein
Glyma20g19250	−	pfkB-like carbohydrate kinase family protein
Glyma20g19470	−	Modifier of rudimentary [Mod(r)] protein
−	Glyma10g24670	P-loop containing nucleoside triphosphate hydrolases superfamily protein
−	Glyma10g24740	Glyma10g24740.1
−	Glyma10g25070	Ferritin 4
−	Glyma10g25340	Plant U-Box 15
Glyma20g19550	Glyma10g25420	Protein of unknown function (DUF3741)
Glyma20g19580	−	Oligosaccharyltransferase complex/magnesium transporter family protein
Glyma20g19600	−	Glyma20g19600.1
Glyma20g19620	−	Glyma20g19620.1
−	Glyma10g25440	Leucine-rich repeat receptor-like protein kinase family protein
Glyma20g19640	Glyma10g25480	GATA transcription factor 1
−	Glyma10g25490	Uncharacterized protein family (UPF0016)
−	Glyma10g25500	Core-2/I-branching beta-1,6-N-acetylglucosaminyltransferase family protein
Glyma20g19670	Glyma10g25510	AT3G22520.1
Glyma20g19680	−	HSP20-like chaperones superfamily protein
Glyma20g19710	−	Hydroxyethylthiazole kinase family protein
−	Glyma10g25550	Hydroxyethylthiazole kinase family protein
Glyma20g19720	Glyma10g25560	Disease resistance-responsive (dirigent-like protein) family protein
Glyma20g19920	−	Disease resistance-responsive (dirigent-like protein) family protein
Glyma20g19930	Glyma10g25560	Disease resistance-responsive (dirigent-like protein) family protein
Glyma20g19940	−	Glyma20g19940.1
−	Glyma10g25570	Disease resistance-responsive (dirigent-like protein) family protein
Glyma20g19970	Glyma10g25620	RING/FYVE/PHD zinc finger superfamily protein
Glyma20g19980	Glyma10g25630	Heat shock protein 60
−	Glyma10g25640	AT5G41980.1
Glyma20g20010	Glyma10g25670	GRIM-19 protein
−	Glyma10g25680	HVA22 homologue E
Glyma20g20040	Glyma10g25690	BLISTER
Glyma20g20050	Glyma10g25700	BLISTER
Glyma20g20070	Glyma10g25710	Coenzyme F420 hydrogenase family/dehydrogenase, beta subunit family
−	Glyma10g25750	AT2G29880.1
Glyma20g20180	Glyma10g25760	YUCCA 3
Glyma20g20280	Glyma10g25760	YUCCA 3
Glyma20g20300	−	Protein kinase superfamily protein
−	Glyma10g25790	Beta-1,2-N-acetylglucosaminyltransferase II
−	Glyma10g25800	Disease resistance family protein / LRR family protein
−	Glyma10g26100	Glyma10g26100.1
−	Glyma10g26120	ROTUNDIFOLIA like 8
−	Glyma10g26150	Ubiquitin C-terminal hydrolase 3
−	Glyma10g26160	Disease resistance family protein/LRR family protein

* *Indicates no G. max gene presents on the chromosome*.

**Figure 2:**
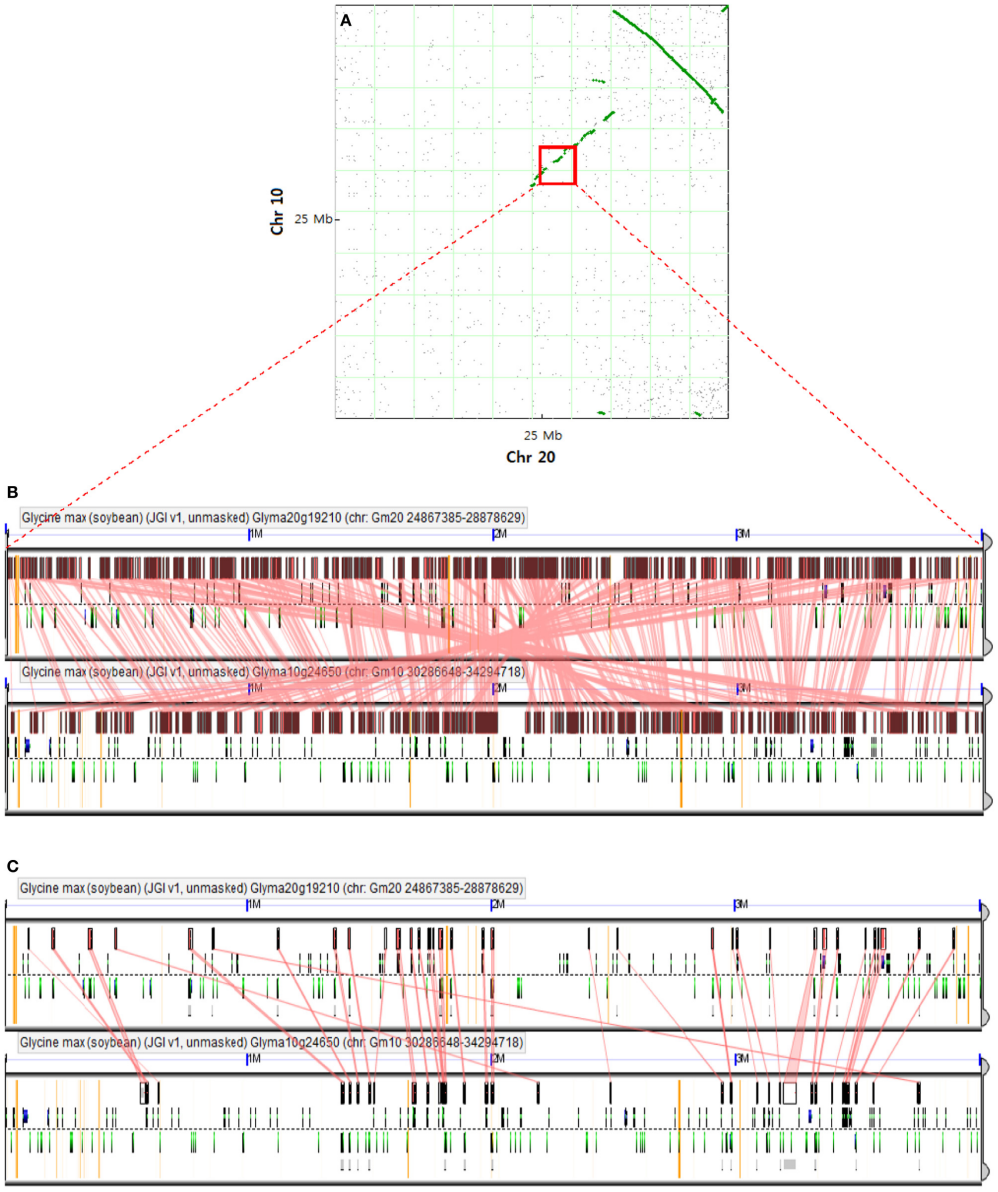
**Comparison of two soybean chromosomes. (A)** Syntenic dot plot of soybean Chr 10 and Chr 20 [adapted from Cannon and Shoemaker (2012)]. Each dot represents homology of the predicted synteny between two chromosomes. A large inversion of synteny in the upper right quadrant is indicated by a line of homology dots that slope down to the right. A positive correlation based on synteny between two chromosomes is denoted by a discontinuous line with a slope moving up to the right. Red square denotes a determined region for alignment algorithm analysis. **(B)** The conserved blocks in the recently duplicated regions of Chr 10 and Chr 20 are based on the alignment of large genomic regions using LastZ algorithm, which is available at http://genomevolution.org/r/56zj. **(C)** Conserved genes in the recently duplicated regions of Chr 10 and Chr 20 based on predicted transcripts using Genome Threader algorithm (spliced alignment of peptides) at http://genomevolution.org/r/56zh.

A large inversion with synteny in the corresponding regions of Chr 20 and Chr 10 was detected by a dot plot comparison between these two Chrs (Figure 2A; Cannon et al., 2004). A positive linear synteny is also observed with a slight interruption (Figure 2A) and leads to survey the conserved blocks along with conserved genes (Figures 2B,C), showing a higher level of synteny with one another. Schmutz et al. (2010) suggested that most of the duplicated regions were conserved but interspersed with insertions/deletions and inversions. All of the syntenic blocks were conserved and some of the syntenic regions between Chr 20 and Chr 10 still obtained a few syntenic genes (Figure 2C), which may reflect the recent genome duplication event regarding gene content (Pagel et al., 2004; Cannon and Shoemaker, 2012).

Among the 19 genes present only on Chr 20, we identified the four tandem duplicates of homeobox protein 22 (HB22), which is reported as *Medicago truncatula* homolog expressed in endosperm at seed filling stage (Verdier et al., 2008). This previous report raises a possibility that these tandem duplicates could regulate the stage of seed filling in soybean and contribute the protein/oil QTL on Chr 20. In addition, several candidate genes identified by Soy GeneChip and transcriptome analyses are thought to be associated with protein content, which may help us understand soybean seed protein regulation, and ten genes were differentially expressed between NILs carrying high and low seed protein content alleles (Bolon et al., 2010).

## Summary

The accumulated genomic data can be used to identify functional genes of specific traits. Even this can provide a basis for predicted gene duplicates following modes of recent duplications. Here, in this review, we compared duplicated genomic regions, which are involved in seed protein content. Increased divergence after recent duplications resulted in the appearance or disappearance of QTLs related to protein and/or oil, suggesting gene retention/loss. Comparing gene and sequence divergence between recently duplicated genomic regions harboring a major QTL for seed protein content on Chr 20, 27 out of 81 genes were present in the homeologous regions of both Chr 20 and Chr 10. Several genes with over- and/or under- retained may be functional and contribute to seed protein content regulation. Therefore, the information of recently duplicated and diverged genes will provide insights into the identification of candidate genes of agronomically important trait.

### Conflict of interest statement

The authors declare that the research was conducted in the absence of any commercial or financial relationships that could be construed as a potential conflict of interest.
